# Antiplasmodial, Antioxidant, and Cytotoxic Activity of *Bridelia micrantha* a Cameroonian Medicinal Plant Used for the Treatment of Malaria

**DOI:** 10.1155/2023/1219432

**Published:** 2023-04-11

**Authors:** Tako Djimefo Alex Kevin, Yamssi Cedric, Noumedem Anangmo Christelle Nadia, Ngouyamsa Nsapkain Aboubakar Sidiki, Mounvera Abdel Azizi, Gamago Nkadeu Guy-Armand, Tientcheu Noutong Jemimah Sandra, Mbohou Nchetnkou Christian, Essangui Same Estelle Géraldine, Tankoua-Tchounda Roméo, Vincent Khan Payne, Lehmann Léopold Gustave

**Affiliations:** ^1^Department of Animal Organisms, Faculty of Science, University of Douala, P.O. Box 24157, Douala, Cameroon; ^2^Department of Biomedical Sciences, Faculty of Health Sciences, University of Bamenda, P.O. Box 39 Bambili, Cameroon; ^3^Department of Microbiology, Hematology and Immunology Faculty of Medicine and Pharmaceutical Sciences, University of Dschang, P.O. Box 96, Dschang, Cameroon; ^4^Department of Animal Biology, Faculty of Science, University of Dschang, P.O. Box 067, Dschang, Cameroon; ^5^Department of Biological Sciences Faculty of Medicine and Pharmaceutical Sciences, University of Douala, P.O. Box 02701, Douala, Cameroon

## Abstract

**Introduction:**

Resistance to common antimalarial drugs and persistence of the endemicity of malaria constitute a major public health problem in Cameroon. The aim of this study was to evaluate the *in vitro* antiplasmodial, antioxidant, and cytotoxic activities of aqueous and ethanol extracts of *Bridelia micrantha* used by Cameroonian traditional healers for the treatment of malaria.

**Methods:**

Aqueous and ethanolic stem bark extracts were prepared according to standard procedures. The SYBR Green method was used for antiplasmodial activity on strains of *Plasmodium falciparum* sensitive to chloroquine (3D7) and resistant (Dd2). *In vitro* antioxidant activities of *B. micrantha* were determined using the scavenging activity of 2,2′-diphenyl-1-picrylhydrazyl, nitric oxide, ferric reducing power, and hydrogen peroxide as well as their cytotoxicity on RAW 264.7 macrophage cells and red blood cells (RBC).

**Results:**

The aqueous and ethanol extracts of *Bridelia micrantha* showed antiplasmodial activity on the 3D7 strain with IC_50_ of 31.65 ± 0.79 *μ*g/ml and 19.41 ± 2.93 *μ*g/ml, respectively, as well as 37.64 ± 0.77 *μ*g/ml and 36.22 ± 1.04 *μ*g/ml for the Dd2 strain, respectively. The aqueous and ethanol extracts showed free radical scavenging properties. The IC_50_ aqueous and ethanol extract was approximately 0.0001737 *μ*g/ml, 42.92 *μ*g/ml, 1197 *μ*g/ml, 63.78 *μ*g/ml and 4.617 *μ*g/ml, 429.9 *μ*g/ml, 511 *μ*g/ml, and 69.32 *μ*g/ml for DPPH, NO, H2O2, and FRAP, respectively, which were compared to ascorbic acid (8.610*e* − 005 *μ*g/ml, 2901 *μ*g/ml, 3237 *μ*g/ml, and 18.57 *μ*g/ml). The aqueous and ethanol extracts of *B. micrantha* were found to be nontoxic with CC_50_ values of 950 ± 6.6 *μ*g/ml and 308.3 ± 45.4 *μ*g/ml, respectively. Haemolysis test showed that the two extracts were not toxic.

**Conclusion:**

These results suggest that *B. micrantha* can serve as an antimalarial agent. However, further studies are needed to validate the use of *B. micrantha* as an antimalarial.

## 1. Introduction

Malaria is caused by a protozoan of the genus *Plasmodium* that is transmitted to humans by the bite of an infected female anopheles mosquito [1]. It is characterised by fever and haemolytic erythrocytopathy due to its presence and development in the liver and red blood cells (RBCs). Malaria is one of the most widespread parasitic diseases globally representing one of the deadliest pandemics for countries in sub-Saharan Africa [2]. It is endemic in the tropical and subtropical areas of Africa, Latin America, and South and South-east Asia, constituting a significant global health burden [3] with about 229 million cases reported in 2021 and over 409,000 deaths. Africa remains the most infected continent, with around 213 million cases recorded [3].

Cameroon is among the 18 countries with 90% of deaths caused by malaria, and 71% of its population is living in areas of high transmission [4]. Children under 5 years and pregnant women are the most infected [5, 6]. Malaria is a major threat having a devastating impact on the public health and well-being of Cameroonians [7–9]. According to the Ministry of Public Health, malaria is the leading cause of consultation (26%), hospitalization (46%), deaths (22%), and annual household health budgets (40%) [8].

In the absence of an effective vaccine against malaria, chemotherapy, chemoprophylaxis, and vector control remain the principal means of managing malaria. The adoption of artemisinin-based combination therapies (ACTs) as first-line drugs for more than 15 years has led to a reduction in mortality in tropical and subtropical regions. However, this achievement is seriously threatened by decreased clinical efficacy of artemisinins [10], as well as their high cost. Humans have long exploited nature as a source of food and medicine. Plants are thus the oldest and most important healing resource available to man [11]. The WHO estimates that over 20,000 plants are used for the treatment of malaria around the world [12]. Traditional healers in the West Region of Cameroon use *Bridelia micrantha* to treat malaria, headaches, gastric ulcers, coughs, rheumatism, dysentery, ethnoveterinary medicine, sexually transmitted infections, stomach aches, tapeworms, and diarrhoea [13].

However, the antiplasmodial effect of this plant has not been scientifically evaluated on the different stages of development of *P. falciparum.*

According to Noumedem et al. [14], there is a direct relationship between malaria and oxidative stress. A plasmodial infection leads to the overproduction of reactive oxygen species. These free radicals are not only toxic to the parasite; they are equally toxic to the host organism. Hence, an antiplasmodial remedy with good antioxidant properties will be an advantage. This study therefore reports the antiplasmodial and antioxidant activities of *B. micrantha* used by traditional healers in the Western Region of Cameroon to treat malaria.

## 2. Material and Methods

### 2.1. Collection and Identification

*Bridelia micrantha*'s stem bark was collected in 2021 in Foumban, West Region of Cameroon. The leaves, seeds, and flowers were sent to the National Herbarium of Cameroon for identification with reference number 64129/HNC attributed to the voucher specimen.

### 2.2. Preparation of Plant Extracts

Powder *B. micrantha* (100 grams) using an electrical balance (SF-400) was weighed into a 2-litre container, with an addition of 95% ethanol (one litre) followed by agitation for five minutes and maceration for 72 hours. Whatman paper no. 3 was used to filter the homogenate with the resulting filtrate evaporated at 45°C in an oven [15]. The aqueous extract was prepared by infusion in distilled water at 100°C for three hours, followed by filtration to obtain the filtrate.

### 2.3. *In Vitro* Antiplasmodial Activity

#### 2.3.1. *Plasmodium* Strain Culture

The Trager [16] technique was used with slight modification. Fresh human group O^+^ red blood cells were used to culture the chloroquine-sensitive *Plasmodium falciparum* strain 3D7 and the multiresistant *Plasmodium falciparum* strain Dd2 at 4% hematocrit in complete RPMI medium ((Gibco, UK) supplemented with 25 mM HEPES (Gibco, UK), 0.50% Albumax I (Gibco, USA), 1Xhypoxanthine (Gibco, USA), and 20 *μ*g/ml gentamicin (Gibco, China)) and incubated at 37°C in a humidified incubator consisting of N (92%), CO_2_ (5%), and O_2_ (3%).

#### 2.3.2. Synchronization of the Culture

The parasite cultures that included a majority of the ring stage (>80%) were synchronized at the ring stage before testing antiplasmodial activity. This was done by treating them with 5% (*w*/*v*) sorbitol for 10 minutes [17].

#### 2.3.3. *In Vitro* Test for *P. falciparum* Growth Inhibition Based on Present Full Meaning of the Acronym (SYBR) Green Fluorescence

The *in vitro* antiplasmodial activity was evaluated according to the method described by Smilkstein et al. [18]. Briefly, 10 *μ*l of the various concentrations of extracts, artemisinin, and chloroquine were put in contact into a 96-well microplate titer with 90 *μ*l of the parasite suspension at the ring stage of 2% parasitemia and 1% hematocrit. The plates were then incubated for 72 hours at 37°C in a CO_2_ incubator. The final plant extract concentration ranged from 0.01258 to 200 *μ*g/ml. This experiment was done in triplicates. One hundred microlitres (100 *μ*l) of SYBR Green was added into each well followed by an hour of incubation in the darkness. The result of the antiplasmodial activity was read using an ELISA fluorescence microplate reader (Tecan Infinite M200) at an excitation and emission wavelength of 485 and 538 nm, respectively. The resistance index (RI) was calculated using the formula:

(1) $$\begin{array}{c} \text{RI}=\frac{{\text{IC}}_{50}\text{ of }Plasmodium\;falciparum\text{ Dd}2}{{\text{IC}}_{50}\text{ of }Plasmodium\;falciparum\;3\text{D}7}. \end{array}$$

### 2.4. *In Vitro* Antioxidant Activity of *B. micrantha*

#### 2.4.1. DPPH Antiradical Activity

The antifree radical activity will be measured using DPPH by the method of [19]. In the spectrophotometer cuvettes, 0.5 ml of plant or vitamin C extracts (at different final concentrations: 1, 3, 10, 30, 100, and 300 *μ*g/ml) will be mixed with 0.5 ml of methanol and 0.5 ml of DPPH (0.063 mg/ml). The blank will consist of 1 ml of methanol and 0.5 ml of DPPH. The absorbance will be read at 517 nm before (DOi) and 20 minutes (DOf) after the introduction of the DPPH solution into the reaction medium, respectively. Vitamin C will serve as a positive control and the test will be performed in 3 replicates.

(2) $$\begin{array}{c} \text{Aox}=\frac{{\left(D_{f}-D_{i}\right)}_{\text{control}}–{\left(D_{f}-D_{i}\right)}_{\text{sample}}}{{\left(D_{f}-D_{i}\right)}_{\text{control}}}\times 100. \end{array}$$

#### 2.4.2. Ferric Reducing Power

The procedure described by Bokhari et al. [20] was followed with slight modification. Briefly, 200 *μ*l of plant extracts/vitamin C were added to test tubes together with 0.5 ml of phosphate-buffered solution (200 mM, pH 6.6) and 0.5 ml of potassium ferricyanide solution. The final concentrations of the plant extracts and vitamin C were 1, 3, 10, 30, 100, and 300 g/ml (30 mM). The absorbance was measured at 700 nm, and the mixture will be incubated for 10 minutes of incubation at 37°C. The test was carried out in triplicate.

#### 2.4.3. Nitric Oxide (NO) Inhibition Test (IT)

When oxygen and NO are combined *in vitro* at physiological pH, they create nitrite ions which may be detected by the Griess reaction [21]. Ten milligrams (10 mg) of extract and vitamin C (at a concentration of 1, 3, 10, 30, 100, or 300 g/ml) were dissolved in 3.53 ml of phosphate-buffered saline (pH = 7.4, 10 mH, pKa = 6.9) to obtain a stock solution. Briefly, 1520 *μ*l of sodium nitroprusside (10 mM) was introduced into test tubes containing 180 *μ*l of *Aframomum pruinosum* or vitamin C extract. The mixture was incubated at 25°C for 2 h 30 min. At the end of the incubation, 500 *μ*l of the previous mixture was taken and introduced into spectrophotometer cuvettes, and then, an equivalent volume of 1% sulfanilamide was added. The mixture was homogenized and incubated for 5 min at room temperature in the dark. 500 *μ*l of naphthyl ethylenediamine (NED, 0.1%) was added to the mixture, followed by another 5 minutes of incubation in the darkness. The absorbance was read at 530 nm to measure the production of the chromophore. The scavenging activity was calculated as follows:

(3) $$\begin{array}{c} \text{Aox}=\frac{\text{DOcontrol}-\text{DOsample}}{\text{DOsample}}\times 100. \end{array}$$

#### 2.4.4. Hydrogen Peroxide Scavenging Activity

The procedure described by Ruch et al. [22] was used to assess if plant extracts have the capacity to break down hydrogen peroxide. Briefly, 0.4 ml of extract at various concentrations (1, 3, 10, 30, 100, or 300 g/ml) was promptly added to 0.6 ml of a hydrogen peroxide solution (40 mM) made in phosphate buffer (pH 7.4, 50 mM). The absorbance of the mixture was measured at 230 nm after ten minutes. Ascorbic acid was considered as a positive control.

(4) $$\begin{array}{c} \%{\text{H}}_{2}\text{O}=\frac{\text{absorbance control}-\text{absorbance sample}}{\text{absorbance control}}. \end{array}$$

### 2.5. Cytotoxic Test

#### 2.5.1. Haemolysis Test

The haemolysis test against healthy erythrocytes was carried out according to the method described by Sinha et al. [23]. Five hundred microlitres (500 *μ*l) of a suspension of healthy erythrocytes from fresh O^+^ blood was prepared at 4% hematocrit in incomplete RPMI1640, in the presence of 500 *μ*l plant extracts at different concentrations in the Eppendorf tubes. Under the same conditions, Triton X-100 at 0.5% (for 100% haemolysis) and the erythrocyte suspension in an incomplete culture medium at 4% hematocrit were used as positive control and negative control, respectively. The final concentrations in the test plates varied from 1000 to 62.5 *μ*g/ml (0.5% DMSO) for the extracts and 0.5% for the Triton X-100 in a final volume of 1000 *μ*l. The plates were incubated at 37°C for 3 hours. After incubation followed by centrifugation at 2500 rpm/3 min, the absorbance of the supernatant corresponding to the release of hemoglobin was measured at 540 nm using the Infinite M200 microplate reader (Tecan). The haemolysis rate of the various extracts was estimated using the formula:

(5) $$\begin{array}{c} \text{Percentage haemolysis }\left(\%\right)=\frac{\left(\text{DO sample}-\text{DO negative control}\right)}{\text{DO positive control}}*100, \end{array}$$

where DO sample is the absorbance of sample, DO negative control is the absorbance of negative control, and DO positive control is the absorbance of positive control.

#### 2.5.2. Cytotoxicity Test on RAW 264.7 Cells

The resazurin-based assay [24] was used to test the extract's ability to kill RAW 264.7 cells. Macrophages were seeded at a density of 10^4^ cells per 100 *μ*l of complete media. After a 24-hour incubation at 37°C with 5% CO_2_, test plates were filled with 10 *μ*l of each serially diluted test sample solution (extract) and then incubated for another 48 hours. Ten microlitres of 0.15 mg/ml of resazurin was added to each well and incubated for 4 hours. Cell proliferation was assessed by measuring the absorbance at 540 nm using the plate reader (Tecan Infinite M200). The selectivity index (SI) was calculated according to the following formula:

(6) $$\begin{array}{c} \text{SI}=\frac{{\text{CC}}_{50}\text{ of RAW cells}}{{\text{IC}}_{50}\text{ of }Plasmodium}. \end{array}$$

For this purpose, the regression lines were drawn using the values read from the different inhibition percentages and the decimal logarithm of the extract concentrations [%inhibition = *f*(log*C*)]. The equations of the regression lines of the form *y* = ax + *b* were used. Assuming each time that *y* = 50, we obtain IC_50_ and CC_50_ = 10*x*, where *x* = (50 − *b*)/*a*.

### 2.6. Statistical Analysis

The fluorescence values obtained were used to calculate the percentage inhibition using the Microsoft Excel software. Next, the replicate number averages of the 50% inhibitory concentration (IC_50_) were determined using concentration-response curves obtained by plotting the logarithm of the concentration versus the percentage inhibition using the GraphPad Prism 8 software. *P* < 0.05 was significant.

## 3. Results

### 3.1. *In Vitro Antiplasmodial Activity* of *Bridelia micrantha*

Table 1 shows the IC_50_ of the aqueous and ethanol extracts of *B. micrantha* on chloroquine-resistant (Dd2) and chloroquine-sensitive (3D7) strains. It appears from this table that the ethanol extract was more active than the aqueous extract on the Dd2 and 3D7 strains, with IC_50_ values of 36.22 ± 1.04 g/ml and 19.41 ± 2.93 g/ml, respectively. The aqueous and ethanol extracts had resistance indexes (RI) of 1.18 and 1.9, respectively.

### 3.2. Antioxidant Activity of *B. micrantha*

#### 3.2.1. DPPH Scavenging Activity

**Figure 1:**
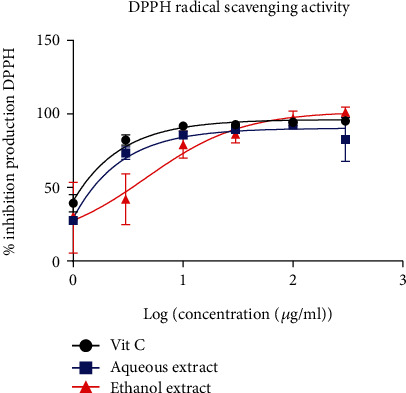
Free radical scavenging of DPPH: % inhibition production according to log (concentration (*μ*g/ml). The data are represented as mean ± standard error of the mean, and each point represents the mean of three replicates.

Figure 1 shows the scavenging activity of *B. micrantha.* The aqueous and ethanol extract have inhibited the production of a DPPH radical. Ascorbic acid showed an IC_50_ of 8.610*e* − 005, whereas the aqueous and ethanol extracts had values of 0.0001737 *μ*g/ml and 4.617 *μ*g/ml, respectively.

#### 3.2.2. Nitric Oxide Free Radical Inhibition

**Figure 2:**
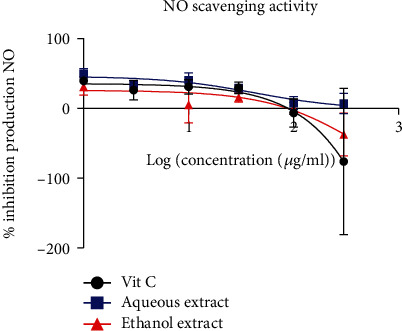
Free radical scavenging of NO: % inhibition production according to log (concentration (*μ*g/ml). The data are represented as mean ± standard error of the mean, and each point represents the mean of three replicates.

The results obtained from the NO production inhibition test are represented in Figure 2. This figure shows that the aqueous and ethanol extracts decrease the production of NO with an increase in the concentration of the extracts. The IC_50_ values of the aqueous extract, ethanolic extract, and ascorbic acid were 42.92, 429.9, and 2901 *μ*g/ml, respectively.

#### 3.2.3. Free Radical Scavenging of Hydrogen Peroxide

**Figure 3:**
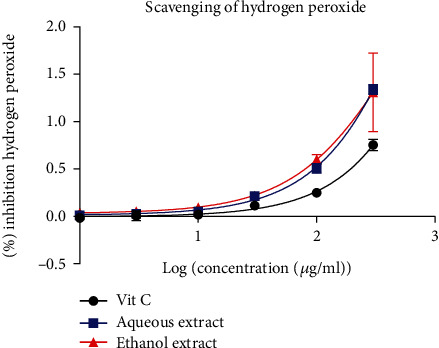
Free radical scavenging of H_2_O_2_: % inhibition hydrogen peroxide according to log (concentration (*μ*g/ml). The data are represented as mean ± standard error of the mean, and each point represents the mean of three replicates.

Figure 3 shows the results obtained for the hydrogen peroxide test of the extracts. The results obtained showed that the standard and extract absorbance increased with respect to concentrations. It emerges from this figure that the aqueous and ethanol extracts inhibited the production of hydrogen peroxide more than ascorbic acid. The IC_50_ of ascorbic acid was 3237 *μ*g/ml which was higher than that of the aqueous and ethanol extracts, with IC_50_ of 1197 and 511 *μ*g/ml, respectively.

#### 3.2.4. FRAP Scavenging Activity

**Figure 4:**
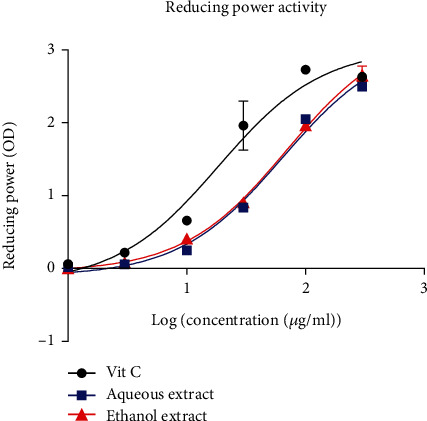
Ferric reducing power activity according to log (concentration (*μ*g/ml). The data are represented as mean ± standard error of the mean, and each point represents the mean of three replicates.

The reducing power activity of the extracts is presented in Figure 4. This figure shows that the aqueous and ethanol extracts of *B. micrantha* demonstrated significant reducing activity. The IC_50_ values of the aqueous and ethanol extracts were 63.78 and 69.32 *μ*g/ml, respectively, and were three times greater than that of ascorbic acid which was found to be 18.57 *μ*g/ml. The results obtained show that the standard and extract absorbances increased as a function of concentrations. Moreover, absorbances of the standard remain higher than that of the extracts.

### 3.3. Cytotoxic Activity of *B. micrantha*

#### 3.3.1. Cytotoxicity on RAW 264.7 Cells

Table 2 presents the results of the cytotoxicity test on RAW 264.7 macrophage cells. It emerges from this table that the aqueous and ethanol extracts had no significant activity on the RAW 264.7 macrophage cells with CC_50_ of 950 ± 6.6 *μ*g/ml and 308.3 ± 45.4 *μ*g/ml for aqueous and ethanol extracts, respectively. The reference drug used was podophyllotoxin with a high CC_50_ of 0.18 ± 0 *μ*g/ml. The selectivity index of the ethanol extract was low (15.9 and 8.51, respectively, of the *Plasmodium* strains 3D7 and Dd2) compared to that of the aqueous extract (30.1 and 25.23, respectively, of the *Plasmodium* strains 3D7 and Dd2).

#### 3.3.2. Haemolysis Test

**Figure 5:**
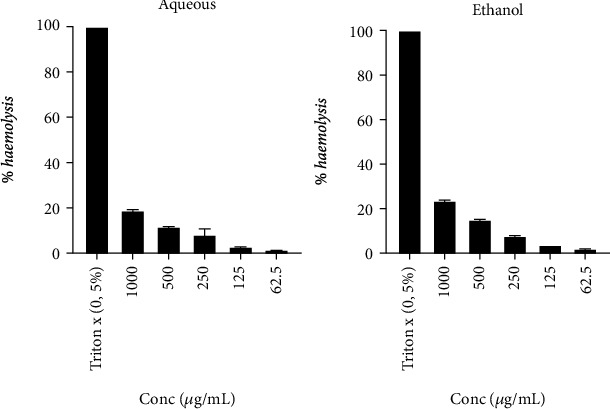
Haemolytic effect of aqueous and ethanol extracts of *B. micrantha.*

The haemolytic activity of plant extracts is expressed as a percentage of haemolysis. The two samples exhibited a very weak haemolytic effect against human erythrocytes. However, these extracts showed a dose-dependent increase in haemolytic activity (Figure 5). The concentrations of 1000 *μ*g/ml and 62.5 *μ*g/ml had an inhibition percentage of 18.86% and 1.52%, respectively.

## 4. Discussion

The results of our investigation showed that the IC_50_ values of the aqueous and ethanolic extracts were 36.22 ± 1.04 *μ*g/ml and 19.41 ± 2.93 *μ*g/ml and 37.64 ± 0.77 *μ*g/ml and 31.65 ± 0.79 *μ*g/ml, respectively, for susceptible Pf3D7 and resistant PfDd2 strains. According to the classification of Kumari et al. [25], when the IC_50_ is higher than 5 *μ*g/ml, the extract is considered very active; when it is between 5 and 50 *μ*g/ml, it is active; when it is between 50 and 100 *μ*g/ml, its activity is moderate; and when it is higher than 100 *μ*g/ml, it is inactive. We can conclude that our aqueous and ethanolic extracts are active on susceptible Pf3D7 and resistant PfDd2 strains. In Nigeria, a similar study was conducted, and the IC_50_ for *B. micrantha* was 158.7 ± 7.82 g/ml [26]. The IC_50_ obtained by Ramadhani et al. [27] was 8 g/ml for *B. micrantha* decoction. The difference in IC_50_ observed may be due to the fact that different strains of *Plasmodium* were used to conduct the experiment. It appears from this study that the ethanolic extract was more active than the aqueous extract. Our results are similar to those obtained by Tomani et al. [28] with *Eriosema montanum* Baker f. roots on *Plasmodium* 3D7 strain which had obtained an IC_50_ of 17.68 ± 4.030 *μ*g/ml of ethanolic extract and different from that obtained by Esseh et al. [29] which had an IC_50_ of 91.08 ± 0.61 *μ*g/ml of aqueous extract. This difference can be explained by the type of extract, and the strain of *Plasmodium* used in this study, as well as the geographical location of the plant, may have had an impact on the observed difference. The resistance index (RI) indicates the inhibitory potential of a drug against sensitive and resistant strains of *P. falciparum.* The resistance index was in the range of 1.18 to 1.9. Extract with RI < 1 is considered promising against sensitive and resistant strains. This shows that the extract of *B. micrantha* had good resistance indices and was classified as interesting and promising.

*Bridelia micrantha* extracts demonstrated strong DPPH free radical scavenging efficacy implying that they possess robust antioxidant properties. The IC_50_ values of the extracts were 0.00017 *μ*g/ml and 4.617 *μ*g/ml for the aqueous and ethanol. A similar study carried out on *Euphorbia neriifolia* of the same family had an IC_50_ of 76.2 ± 0.07 inhibition [30]. This could be justified by the fact that all plants belonging to the same family do not necessarily have the same mode of action. *Bridelia micrantha* extract has the ability to donate protons that can serve as an inhibitor or scavenger of free radicals, acting as a primary [30].

These plant extracts increased the amount of nitrite produced from the breakdown of sodium nitroprusside *in vitro*, suggesting that direct scavenging of the NO radical may not be related to the suppression of its release. The plant extract did not significantly improve the dose-dependent scavenging of nitric oxide. The IC_50_ values for the extracts were 42.92 *μ*g/ml for the aqueous and 429.9 *μ*g/ml for the ethanol extract, while the IC_50_ value of ascorbic acid was 2901 *μ*g/ml. This result suggests that *B. micrantha* extracts would not intercept the NO formation reaction from nitroprusside but would rather act in favor of this reaction. On the other hand, these extracts could be beneficial in the management of arterial hypertension, one of the major characteristics of which is the decrease in the bioavailability of NO. Nitric oxide and superoxide anions have the potential to injure a variety of tissues. Peroxynitrite (ONOO^−^), which is formed when NO and O^2-^ react, worsens the toxicity profile and damages it because it causes toxic interactions with biomolecules [31, 32]. The cascade of reactions triggered by excessive NO formation can be stopped by scavenging the reactive peroxynitrite, which will help stop the negative effects that these radicals have in the human body.

The H_2_O_2_ scavenging activity of the aqueous and ethanolic extracts was slightly higher than the positive control. The IC_50_ values of the extract were 1197 *μ*g/ml and 511 *μ*g/ml for the aqueous and ethanol, while the IC_50_ value of ascorbic acid was 3237 *μ*g/ml. Hydrogen peroxide can produce hydroxyl ions in the presence of iron ions, which can be harmful to cells [33]. Therefore, in order to provide antioxidant defense, H_2_O_2_ must be removed from cells. The reducing power of *B. micrantha* extracts increases as the concentration increases. This is so because reducers break the chain of the free radicals donating a hydrogen atom. Their presence is closely related to the reducing activities of extracts [34]. Since a considerable reducing activity was noticed, it means our plant extracts contain phytochemical constituents capable of reacting with ferric-tripyridyl triazine (Fe^3+^-TPTZ) complex to produce Fe^2+^-TPTZ as the end product.

The CC_50_ values of the extracts in this study were 950 ± 6.6 *μ*g/ml and 308.3 ± 45.4 *μ*g/ml for the aqueous and ethanol extracts, respectively, on RAW 264.7 macrophage cells. According to the cytotoxicity classification of Malebo et al., [35] a CC_50_ < 1.0 *μ*g/ml is high cytotoxicity, CC_50_ < 1.0–10.0 *μ*g/ml is moderate cytotoxicity, CC_50_ < 10.0 − 30 *μ*g/ml is average cytotoxicity, and CC_50_ > 30.0 *μ*g/ml is noncytotoxic. Hence, the extracts from *B. micrantha* can be classified as nontoxic. The aqueous extract had a SI of 30.01 and 25.23 for Pf3D7 and PfDd2, respectively, and was higher than that of the ethanol extract which was 15.9 and 8.51 for Pf3D7 and PfDd2, respectively. A similar study was conducted by Bapela et al. [36] on *B. mollis* Hutch where they had a CC_50_ of 51.4 *μ*g/ml with a SI of 17. The difference observed here may be due to the cell strain used for the cytotoxicity test.

Many researchers have used erythrocytes as a model system to explore the interaction of drugs with membranes [37]. The concentration and potency of the extract are linked to haemolysis. In addition, the chemical composition of each extract affects its haemolytic action. The aqueous and ethanol extracts of *B. micrantha* had no effect and does not affect the erythrocyte membrane at low concentrations, but attention should be given to high doses like 1000 *μ*g/ml.

## 5. Conclusion

The aqueous and ethanol extracts of *B. micrantha* were found to be active against *Plasmodium falciparum*-sensitive 3D7 and -resistant Dd2 strains but nontoxic on RBCs and macrophage cells (RAW 264.7). The results also showed that the aqueous and ethanol extracts of *B. micrantha* have promising antioxidant activity, suggesting that it may be useful in the prevention of many oxidative stress-related diseases. However, studies must be carried out to scientifically confirm the use of *B. micrantha* as an antiplasmodial remedy and to assess its safety.

## Figures and Tables

**Table 1:** Antiplasmodial activity of *B. micrantha* on chloroquine-sensitive (3D7) and Dd2-resistant *Plasmodium* strains from aqueous and ethanolic extracts.

Sample	IC_50_ ± SD (*μ*g/ml)		RI	Observation
	PfDd2	Pf3D7		
Aqueous	37.64 ± 0.77^∗∗^	31.65 ± 0.79	1.18	Active
Ethanol	36.22 ± 1.04	19.41 ± 2.93	1.9	Active
Positive control				
Artemisinin (*μ*M)	0.043 ± 0.0113^∗∗^	0.034 ± 0.0048	1.26	NA
Chloroquine (*μ*M)	0.064 ± 0.083^∗∗^	0.029 ± 0.00037	2.2	NA

Note: ^∗∗^*P* value < 0.01. Legend: IC_50_: inhibitory concentration 50; IR: resistance index; NA: not applicable.

**Table 2:** Cytotoxicity (RAW 264.7) and selectivity index.

Extract	CC_50_ (*μ*g/ml)	SI	
		3D7	Dd2
Aqueous	950 ± 6.6	30.01	25.23
Ethanolic	308.3 ± 45.4	15.9	8.51
Podophyllotoxin (positive control)	0.18 ± 0	NA	NA

NA: not applicable.

## Data Availability

All data generated and analyzed are included in this research article.

## References

[1] Yaméogo J. B. G., Annabelle G., Luc C. (2012). Self-assembled biotransesterified cyclodextrins as Artemisinin nanocarriers – I: Formulation, lyoavailability and in vitro antimalarial activity assessment. *European Journal of Pharmaceutics and Biopharmaceutics*.

[2] Chouto S., Wakponou A. (2017). Disparités spatio-temporelles et prévalence du paludisme à partir des données formelles : cas de Kousséri (Extrême-Nord Cameroun). *Journal of Ecology and the Natural Environment*.

[3] OMS (2021). *Rapport sur le paludisme dans le monde 2020*.

[4] Zofou D., Tene M., Ngemenya M. N., Tane P., Titanji V. P. (2011). _In Vitro_ Antiplasmodial Activity and Cytotoxicity of Extracts of Selected Medicinal Plants Used by Traditional Healers of Western Cameroon. *Malaria Research and Treatment*.

[5] Mbohou C. N., Foko L. P., Nyabeyeu H. N. (2019). Malaria screening at the workplace in Cameroon. *PLoS One*.

[6] Sidiki N. N., Payne V. K., Cedric Y., Nadia N. A. (2020). Effect of impregnated mosquito bed nets on the prevalence of malaria among pregnant women in Foumban subdivision, West Region of Cameroon. *Journal of Parasitology Research*.

[7] Akono P. N., Mbida A. M., Tonga C., Kayoum A. Y., Youmbi L. E., Lehman L. G. (2017). Données préliminaires sur le paludisme humain en zones rurale et sémi-urbaine du département du Nkam (Littoral-Cameroun). *Journal of Applied Biosciences*.

[8] Minsanté (2018). *Plan stratégique de lutte contre le paludisme au Cameroun*.

[9] Antonio-Nkondjio C., Ndo C., Njiokou F. (2019). Review of malaria situation in Cameroon: technical viewpoint on challenges and prospects for disease elimination. *Parasite and Vectors*.

[10] Benoit-Vical F., Paloque L., Augereau J. M. (2016). Plasmodium falciparum resistance to artemisinin-based combination therapies (ACTs): fears of widespread drug resistance. *Bulletin de L'academie Nationale de Medecine*.

[11] Carillon A. (2009). Place de la phytothérapie dans les systèmes de santé au XXI s. *SIPAM*.

[12] Fatimata N. (2021). *Etude phytochimique et biologique de deux plantes médicinales de Côte d’Ivoire: Lantana camara et Lantana rhodesiensis (Verbenaceae)*.

[13] Maroyi A. (2017). Ethnopharmacology and therapeutic value of Bridelia micrantha (Hochst.) baill. In tropical Africa: a comprehensive review. *Molecules*.

[14] Nadia N., Pone J., Arlette N. (2017). In vitro antiplasmodial and antioxidant activities of Entandrophragma cylindricum (Meliaceae) extracts. *European Journal of Medicinal Plants*.

[15] Ciulei I. (1982). *Practical manuals on the industrial utilization of chemical and aromatic plants. Methodology for analysis of vegetable drugs*.

[16] Trager W., Jensen J. B. (1976). Human malaria parasites in continuous culture. *Science*.

[17] Lambros C., Vanderberg J. P. (1979). Synchronization of Plasmodium falciparum erythrocytic stages in culture. *The Journal of Parasitology*.

[18] Smilkstein M., Sriwilaijaroen N., Kelly J. X., Wilairat P., Riscoe M. (2004). Simple and inexpensive fluorescence-based technique for high-throughput antimalarial drug screening. *Antimicrobial Agents and Chemotherapy*.

[19] Popovici C., Saykova I., Tylkowski B. (2009). Evaluation de l'activité antioxydant des composés phénoliques par la réactivité avec le radical libre DPPH. *Revue de Génie Industriel*.

[20] Bokhari J., Muhammad R. K., Maria S., Umbreen R., Shumaila J., Jawaid A. Z. (2013). Evaluation of diverse antioxidant activities of _Galium aparine_. *Spectrochimica Acta Part A: Molecular and Biomolecular Spectroscopy*.

[21] Ashokkumar D., Thamilselvan V., Mazumder U. K., Gupta M. (2008). Antioxidant and free radical scavenging effects ofLippia nodiflora. *Pharmaceutical Biology*.

[22] Ruch R. J., Crist K. A., Klaunig J. E. (1989). Effects of culture duration on hydrogen peroxide-induced hepatocyte toxicity. *Toxicology and Applied Pharmacology*.

[23] Sinha S., Batovska D. I., Medhi B. (2019). In vitro anti-malarial efficacy of chalcones: cytotoxicity profile, mechanism of action and their effect on erythrocytes. *Malaria Journal*.

[24] Bowling T., Mercer L., Don R., Jacobs R., Nare B. (2012). Application of a resazurin-based high-throughput screening assay for the identification and progression of new treatments for human African trypanosomiasis. *International Journal for Parasitology: Drugs and Drug Resistance*.

[25] Kumari S., Satish P., Somaiah K., Rekha S., Brahmam P., Sunita K. (2016). Antimalarial activity of Polyalthia longifolia (false ashoka) against chloroquine sensitive Plasmodium falciparum 3D7 strain. *World Journal of Pharmaceutical Sciences*.

[26] Edith A., Joseph A., Oyindamola A. (2004). Antimalarial ethnobotany: in vitro antiplasmodial activity of seven plants identified in the Nigerian Middle Belt. *Pharmaceutical Biology*.

[27] Nondo R. S., Zofou D., Moshi M. J. (2015). Ethnobotanical survey and in vitro antiplasmodial activity of medicinal plants used to treat malaria in Kagera and Lindi regions, Tanzania. *Journal of Medicinal Plants Research*.

[28] Tomani J. C., Bonnet O., Nyirimigabo A. (2021). In vitro antiplasmodial and cytotoxic activities of compounds from the roots of Eriosema montanum Baker f. (Fabaceae). *Molecules*.

[29] Esseh K., Afanyibo Y. G., Ahama-Esseh K. Y. (2019). Screening Phytochimique, Étude Toxicologique, Évaluation des Activités Antiplasmodiale et Antiradicalaire de la Tige Feuillée de Senna occidentalis Linn (Fabaceae). *European Scientific Journal*.

[30] Chaudhary P., Janmeda P. (2022). Quantification of phytochemicals and in vitro antioxidant activities from various parts of Euphorbia neriifolia Linn. *Journal of Applied Biology and Biotechnology*.

[31] Halliwell B., Whiteman M. (2004). Measuring reactive species and oxidative damage in vivo and in cell culture: how should you do it and what do the results mean?. *British Journal of Pharmacology*.

[32] Zhou J., Wu P., Wang F., Chen J. (2012). Targeting gaseous molecules to protect against cerebral ischaemic injury: mechanisms and prospects. *Clinical and Experimental Pharmacology and Physiology*.

[33] Jagetia G. C., Reddy T. K. (2011). Alleviation of iron induced oxidative stress by the grape fruit flavanone naringin in vitro. *Chemico-biological interactions*.

[34] Viuda-Martos M., Ruiz-Navajas Y., Fernández-López J., Sendra E., SayasBarberá E., Pérez-Álvarez J. A. (2011). Antioxidant properties of pomegranate (_Punica granatum_ L.) bagasses obtained as co-product in the juice extraction. *Food Research International*.

[35] Malebo H. M., Tanja W., Cal M. (2009). Antiplasmodial, antitrypanosomal, anti-leishmanial and cytotoxicity activity of selected Tanzanian medicinal plants. *Tanzania Journal of Heath Research*.

[36] Bapela J., Heyman H., Senejoux F., Meyer M. (2019). ^1^H NMR-based metabolomics of antimalarial plant species traditionally used by Vha-Venda people in Limpopo Province, South Africa and isolation of antiplasmodial compounds. *Journal of Ethnopharmacology*.

[37] Costa-Lotufo L., Khan M., Ather A. (2005). Studies of the anticancer potential of plants used in Bangladeshi folk medicine. *Journal of Ethnopharmacology*.

